# Top 10 research priorities for sepsis research determined by patients, carers and clinicians

**DOI:** 10.1111/anae.16634

**Published:** 2025-06-05

**Authors:** Joanne McPeake, Nahid Ahmad, Kimberley Bradley, Andrew Conway Morris, Paul Dark, Colin Graham, Walter Hall, Susan Moug, Mark Oakes, Emily Perry, Simon Stockley, Jane Weaver, Bronwen Connolly, Nazir Lone

**Affiliations:** ^1^ The Healthcare Improvement Studies Institute University of Cambridge Cambridge UK; ^2^ The James Lind Alliance University of Southampton Southampton UK; ^3^ Patient Representative UK; ^4^ Division of Perioperative, Acute, Critical Care and Emergency Medicine, Department of Medicine University of Cambridge Cambridge UK; ^5^ John V Farman Intensive Care Unit, Addenbrooke's Hospital Cambridge UK; ^6^ Division of Immunology, Immunity to Infection and Respiratory Medicine Faculty of Biology, Medicine and Health, Critical Care Unit, Northern Care Alliance NHS Foundation Trust, Salford Care Organisation Greater Manchester UK; ^7^ Sepsis Research (FEAT) Glasgow UK; ^8^ Department of Surgery Royal Alexandra Hospital Paisley UK; ^9^ College of Medical, Veterinary, and Life Sciences, University of Glasgow UK; ^10^ Eaglescliffe Medical Practice Eaglescliffe UK; ^11^ Wellcome‐Wolfson Institute for Experimental Medicine, Queen's University Belfast Belfast UK; ^12^ Department of Physiotherapy The University of Melbourne Melbourne Australia; ^13^ The Usher Institute, University of Edinburgh Edinburgh UK

**Keywords:** consensus, James Lind Alliance, priority, research, sepsis

## Abstract

**Introduction:**

Sepsis is a high burden syndrome associated with increased morbidity and mortality in both the acute and longer‐term phases of illness. Multiple treatment uncertainties remain that require resolution through high‐quality research. This study aimed to identify the top 10 research priorities for sepsis research in the UK.

**Methods:**

We conducted a priority setting partnership study co‐produced by sepsis survivors, carers and clinicians. This included five stages: initiation of steering group formation and confirmation of the scope of the priority setting partnership; identification of clinical uncertainties through an electronic survey; analysis and verification of uncertainties; interim prioritisation to the top 25 ranked questions; and final prioritisation to determine the top 10 research priorities, using the nominal group technique.

**Results:**

Our initial survey respondents comprised 447/718 (62.3%) people who had survived sepsis, their friends and family members; 218/718 (30.4%) clinicians; and 53/718 (7.1%) multiple/other roles who identified 53 distinct research uncertainties. Our interim prioritisation survey comprised 429/941 (45.8%) people who had survived sepsis, their friends and family members; 431/941 (46.0%) clinicians; and 73/941 (8.2%) multiple/other roles, with the top 25 ranked summary questions taken forwards for final prioritisation. From these, final workshop participants (n = 27) agreed a top 10 list of research priorities. Improved sepsis diagnosis; characterisation and management of the post‐sepsis syndrome; and non‐antibiotic treatment of sepsis were the top three priorities.

**Discussion:**

We established priorities for sepsis research through a rigorous process of consensus involving sepsis survivors, carers and clinicians. These priorities will support future delivery of meaningful research to improve outcomes from sepsis.

## Introduction

Sepsis is a global healthcare problem that impacts all age groups. Pre‐pandemic data suggest that sepsis kills over 45,000 people each year in the UK, with estimated annual NHS costs of £1.1 billion ($1.5 billion, €1.3 billion) and wider societal costs of up to £10 billion ($13 billion, €12 billion) [1]. In addition to substantial acute mortality, sepsis has long‐term consequences. The prognosis for sepsis survivors after hospitalisation can manifest in long‐term physical, emotional, social and cognitive issues [2, 3], commonly referred to as post‐sepsis syndrome. Readmissions to hospital following a sepsis episode are common, with sepsis survivors at increased risk of long‐term mortality [4].

Despite the overwhelming short‐ and long‐term burden of sepsis, there remain many treatment uncertainties (questions that cannot be answered by existing research [5] and are yet to be clarified through well designed and adequately powered clinical trials and research [6]). However, research priorities from the perspectives of investigators often fail to align with those of patients and carers and the healthcare professionals responsible for delivering their care [5]. The UK National Institute for Health and Care Research (NIHR) supports patient and public involvement and the ‘co‐production’ of research. However, there has been no systematic and inclusive dialogue between clinicians, patients and carers to identify sepsis research priorities.

The James Lind Alliance (JLA) facilitates priority setting partnerships to foster co‐production dialogue and aims to engage those with lived experience of a disorder, carers and clinician groups to identify research uncertainties that are important to all groups in a particular area of health. Research uncertainties are prioritised subsequently to ascertain the top 10 research questions to underpin a future research agenda to address these questions. To understand the priorities of survivors of sepsis, carers and clinicians, we conducted a JLA priority setting partnership in sepsis research to identify the key treatment research uncertainties for the sepsis community.

## Methods

This study followed the UK JLA methodology [7]. This robust approach to the development of key research priorities is described elsewhere [8, 9]. A short summary of the project has been published previously [10]. This current scientific report details the full methodology and the full list of research priorities.

The three clinical leads of the project (JM, BC and NL) submitted a readiness questionnaire which was approved by the JLA (NIHR, School of Healthcare Enterprise and Innovation, University of Southampton, UK). The JLA facilitated provision of an independent chair (NA) to simplify the process and ensure that JLA methodology was adhered to appropriately and robustly. We sought advice from our JLA advisor regarding ethical review before starting and concluded that, in line with other JLA priority setting partnerships, this was not required. Participants provided informed consent (indicated by survey completion and agreement to workshop attendance); it was made clear at each stage of the priority setting partnership that participation was voluntary, what participation involved, the purpose of the study and the use of data. The Checklist for Reporting of Survey Studies was used to report findings [11].

Through peer knowledge and consultation, we formed a steering group for the Sepsis Research priority setting partnership. The steering group was responsible for: agreement on the initial focus; review of priority setting partnership documents and consultation tools; publicity of the priority setting partnership across relevant professional and public organisations; and oversight and collation of the identified priorities. Steering group meetings were chaired by the JLA advisor (NA) and included patients and family members with lived experience of sepsis (KB, WH, EP, JW and MO). Sepsis can be diagnosed and managed across the entire healthcare system; as such, a diversity of experience was sought for the steering group, including general practitioners (SS); critical care practitioners (JM, NL, BC and ACM); emergency physicians (PD) and surgeons (SM). At the initial steering group meeting, the scope of the Sepsis Research priority setting partnership was confirmed. This included all aspects of the management of sepsis in adults, focusing on susceptibility, diagnosis, diagnostic engineered tools, treatment (any therapy in hospital and longer‐term) and outcomes (physical, psychological, social and societal), including assessing impact and improving outcomes. In addition to patients and clinicians, we explored the perspectives of family members and caregivers. Our scope did not include questions about the precursor infection leading to sepsis; the paediatric population; and family members who were bereaved from sepsis. The full priority setting partnership protocol was published on the JLA website [12].

We conducted the first consultation exercise to understand uncertainties related to sepsis research via an initial electronic survey. The survey was launched in September 2023, coinciding with World Sepsis Day and remained open until January 2024.

Survey participants were asked initially to identify three uncertainties or unanswered questions that they wished to see addressed by future sepsis research. Additional optional questions included demographic details (sex, age range (collected in deciles) and ethnicity); name and preferred contact email; and the respondent category that best described their interest in participating (sepsis survivor, family member or carer of someone who has had sepsis, clinician/care provider, multiple roles or other (including third sector personnel, researcher and government/policy)). Contact details were collected only for the purposes of inviting participation to future activities related to the priority setting partnership and survey participants could remain anonymous.

The survey was promoted through multiple forums to leverage the collective resource of the steering group. These included clinical and patient networks; professional organisations and colleges; social media (Facebook and X); national broadcasting (television and radio); and the Sepsis Research FEAT website. Additionally, we engaged with the Centre for Research Equity (University of Oxford, UK) to promote the priority setting partnership in under‐represented communities.

All questions submitted from the online survey were assigned a unique question code. These were reviewed by an independent information specialist and thematically grouped into categories. Duplicate questions and those deemed out of scope were removed. The remaining questions were grouped into summary questions. A sub‐section of the steering group, which included those with lived experience and healthcare professionals, reviewed the summary questions to ensure these were representative of the original questions. Each summary question was then searched against the existing literature to ensure that the question was not already answered by previous research. Members of the steering group then agreed on the summary questions to be put forward for interim prioritisation based on being representative of the wider questions submitted and not answered by existing research.

A second survey was distributed between March and June 2024 using the same dissemination routes as the initial survey. Survey participants were asked to identify the top 10 questions they felt to be most important from a list of all summary questions presented randomly. Following closure of the survey in June 2024, the resulting highly ranked (based on frequency chosen) 25 questions were taken forward for final prioritisation. The source of each question was reviewed to ensure that questions from those with lived experience and clinicians were represented in the combined top 25 (lived experience 17/25 (68%) taken forward; clinicians 18/25 (72%) taken forward).

The top 25 questions identified after the second survey were then taken forward to a final face‐to‐face prioritisation workshop. This took place in September 2024 and was chaired by NA with support from two additional JLA advisors. Participants had expressed their interest in taking part by submitting their contact details in either survey. During the workshop, attendees were divided into three groups, with an equal mix of people with lived experience and clinicians in each. Each group was facilitated by a JLA advisor to ensure all participants were able to voice their opinions. Groups were asked to rank the 25 questions through structured discussions. Rankings across the three groups were then combined, the groups mixed and the questions ranked a second time; this produced a final combined rank order. All workshop attendees then discussed the results collectively and confirmed the final ranking with a focus on the top 10 prioritised research uncertainties.

Patients and family members were involved as equal members of the steering group at all stages of the priority setting partnership process. People with lived experience of sepsis were equal partners in the final prioritisation workshop.

## Results

**Figure 1:**
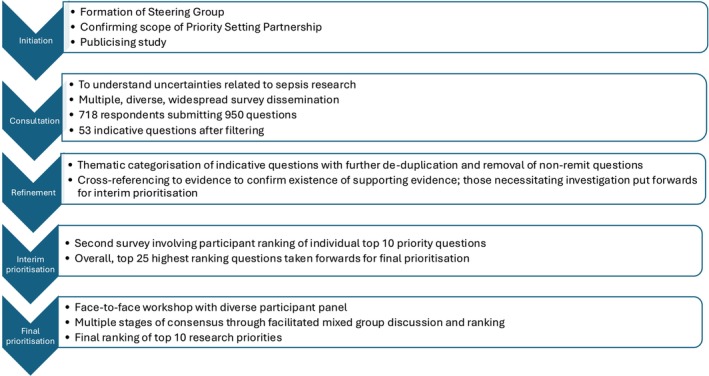
Summary of the priority setting partnership process.

A summary of the priority setting partnership process is shown in Figure 1. The initial survey was answered by 718 individual respondents (where data provided: female 250/344 (72.7%); age 45–64 y, 171/348 (49.1%); and White ethnicity, 316/338 (93.5%)). Our initial survey respondents comprised 447/718 (62.3%) who had survived sepsis, their friends and family members; 218/718 (30.4%) clinicians; and 53/718 (7.1%) multiple/other roles (Table 1). In total, 950 questions were submitted from this initial survey. Removal of duplicate and out‐of‐scope responses and subsequent thematic grouping by the independent information specialist resulted in 53 summary questions that had not been answered within the current evidence base. The information specialist also led the evidence review, which was conducted between January and March 2024.

**Table 1:** Characteristics of survey participants. Values are number (proportion), calculated after exclusion of missing data.

Characteristics	Survey 1 – initial survey, n = 718	Survey 2 – interim prioritisation survey, n = 941	Final workshop, n = 27
**Age; y**			
16–24	13 (3.7%)	9 (1.4%)	1 (3.7%)
25–34	39 (11.2%)	79 (12.6%)	2 (7.4%)
35–44	57 (16.4%)	124 (19.8%)	4 (14.8%)
45–54	94 (27.0%)	176 (28.1%)	12 (44.4%)
55–64	77 (22.1%)	131 (20.9%)	6 (22.2%)
65–74	55 (15.8%)	70 (11.2%)	2 (7.4%)
75–84	13 (3.7%)	32 (5.1%)	0
85–94	0	5 (0.8%)	0
Missing	370 (51.5%)	315 (33.4%)	0
**Sex at birth**			
Female	250 (72.7%)	407 (65.2%)	13 (50%)
Male	94 (27.3%)	217 (34.8%)	13 (50%)
Missing	374 (52.0%)	317 (33.6%)	1 (3.7%)
**Ethnicity**			
White	316 (93.5%)	584 (94.8%)	23 (92.0%)
Asian	9 (2.7%)	17 (2.8%)	1 (4.0%)
Black	9 (2.7%)	5 (0.8%)	
Mixed	< 5 (< 2.0%)	6 (1.0%)	
Other	< 5 (< 2.0%)	4 (0.6%)	1 (4.0%)
Missing	380 (52.9%)	325 (34.5%)	2 (7.4%)
**Sexual orientation**			
Heterosexual	313 (93.7%)	557 (95.2%)	
Bisexual/pansexual/asexual/other	13 (3.9%)	14 (2.4%)	
Homosexual	8 (2.4%)	14 (2.4%)	
Missing	384 (53.4%)	356 (37.8%)	
**Respondent category**			
Clinician or care provider	218 (30.4%)	431 (46.0%)	14 (51.9%)
Sepsis survivor	256 (35.7%)	268 (28.6%)	12 (44.4%)
Family member or carer	191 (26.6%)	161 (17.2%)	1 (3.7%)
Multiple roles	48 (6.7%)	65 (6.9%)	0
Other role	5 (0.4%)	12 (1.3%)	0
Missing	0	4 (0.43%)	0

The interim prioritisation survey was answered by 941 respondents (where data provided: female 407/624 (65.2%); age 45–64 y, 307/626 (49.0%); and White ethnicity 584/616 (94.8%)). Compared with the initial survey, respondent categories were more evenly spread between clinicians (431/937 (46.0%)) and sepsis survivors and family members/carer (429/937 (45.8%)), with 77/937 respondents (8.2%) citing multiple/other roles.

The top 25 ranked summary questions from the second survey taken forward for final prioritisation at the workshop are listed in Table 2. Workshop participants (n = 27) were representative of geographical diversity, ethnicity and age, and included healthcare professionals as well as those with lived experience. Workshop participant characteristics are provided in Table 1 and the results of the final top 10 priorities for sepsis research are listed in Table 3.

**Table 2:** Top 25 ranked summary questions taken forwards for final prioritisation.

Ranking	Question
1	How can the diagnosis of sepsis become faster, more accurate and reliable?
2	Can diagnostic tests be developed for sepsis that can be used wherever the person is receiving care (e.g. in a GP surgery, hospital, ambulance or at home)?
3	How often is sepsis genuinely missed? Why does this happen? What would help health professionals to recognise sepsis (e.g. additional training)?
4	Would treatment before admission to hospital, (e.g. provided by GPs or ambulance crews), improve outcomes for people with sepsis?
5	Why and how do some people with sepsis become seriously ill very quickly?
6	What impact is Sepsis Six (a set of six steps to be taken within an hour of sepsis diagnosis) having on the recognition and treatment of sepsis?
7	Does sepsis affect the immune system in the long‐term, increasing the risk of infections and/ or autoimmune conditions? If yes, how is this best treated and managed?
8	How does an infection lead to sepsis?
9	What happens during sepsis to cause long‐term effects on the body (sometimes called post‐sepsis syndrome)?
10	What are the long‐term effects on the body from sepsis (sometimes called post‐sepsis syndrome)? How are these long‐term effects best treated and managed?
11	What are the barriers to people with sepsis receiving rapid treatment once diagnosed, and how can these be overcome?
12	Are there ways to tailor treatment of sepsis to the individual (e.g. based on blood markers or other indicators)?
13	Would specialist sepsis services improve outcomes for people with sepsis during hospital treatment and for follow‐up care?
14	What is the role of treatments other than antibiotics in the care and management of sepsis?
15	Does sepsis increase the risk of developing long term conditions (e.g. heart disease and chronic kidney disease)? How are these conditions best treated?
16	Can a test be developed to predict the outcome of sepsis, to identify which people are likely to recover or become seriously ill or die?
17	Are there ways to prevent long‐term effects on the body (sometimes called post‐sepsis syndrome) through treatment while the person has sepsis?
18	What are the safest and most effective ways to treat sepsis using antibiotics?
19	Are some people at greater risk of developing sepsis because of their genetic make‐up? Does this mean their family members are also at risk?
20	What are the best ways to monitor people after sepsis and to provide follow‐up care?
21	What factors (e.g. genetics, age or ethnic background) influence whether a person recovers or dies from sepsis, and how well a person recovers?
22	How can communication and care co‐ordination be improved across the teams of health professionals caring for people with sepsis?
23	Are there any aspects of intensive care/ hospital treatments that increase the risk of becoming seriously ill with sepsis?
24	How likely are people to experience repeat episodes of sepsis? Are repeat episodes different to the first experience?
25	Does living in poverty increase the risk of developing sepsis and/or lead to worse outcomes? If yes, how and why does this happen?

**Table 3:** Top 10 research priorities for sepsis.

Ranking	Question
1	How can the diagnosis of sepsis become faster, more accurate and reliable?
2	What are the long‐term effects on the body from sepsis (sometimes called post‐sepsis syndrome)? How are these long‐term effects best treated and managed?
3	What is the role of treatments other than antibiotics in the care and management of sepsis?
4	Can diagnostic tests be developed for sepsis that can be used wherever the person is receiving care (e.g. in a GP surgery, hospital, ambulance or at home)?
5	Why and how do some people with sepsis become seriously ill very quickly?
6	Would specialist sepsis services improve outcomes for people with sepsis during hospital treatment and for follow‐up care?
7	Are there ways to tailor treatment of sepsis to the individual (e.g. based on blood markers or other indicators)?
8	How does an infection lead to sepsis?
9	Would treatment before admission to hospital (e.g. provided by GPs or ambulance crews) improve outcomes for people with sepsis?
10	What are the safest and most effective ways to treat sepsis using antibiotics?

## Discussion

We have developed priority research questions for sepsis in the UK, co‐produced through a priority setting partnership inclusive of patients, carers and clinicians. The top 10 priorities provide a scaffold to inform commissioned and researcher‐led funding decisions from both government and charitable organisations. Notably, the top‐ranked priority, “*How can the diagnosis of sepsis become faster, more accurate, and reliable?*” featured highest across the respondent groups during the priority setting partnership process in the shortlisting survey, and as the top‐ranked question throughout the workshop by all three discussion groups. This reflects the shared importance of continuing to improve timely and rigorous identification of sepsis. Overall, the top 10 priorities reflect the breadth of the sepsis experience spanning the trajectory of diagnosis, treatment and long‐term impact and survivorship. Priorities highlight the need for research strategies to be holistic in their approach rather than driven solely by biological, mechanistic and drug therapies. Funders and researchers now have the mandate to align future investigation on what is important to survivors, families and professionals in the UK.

Previous exploration of research priorities for sepsis has been conducted, albeit focusing on survivorship and long‐term recovery and utilising different methodology. Prescott et al. used a nominal group technique within a structured invited colloquium setting to gain consensus rapidly within a panel of international multidisciplinary clinicians and researchers [13]. Agreed priorities tended to focus on broader methodological approaches, rather than specific research questions as generated in our priority setting partnership. For example, in the shorter‐term, focusing on international data merging and harmonisation; provision of educational resources; and partnership with survivor groups. Longer‐term priorities included a global cohort study to determine mechanisms of long‐term outcomes; a global sepsis registry; and phenotyping variable sepsis recovery through detailed long‐term follow‐up [13]. Aligning both research prioritisation exercises, the value of the current priority setting partnership findings is to clearly articulate research questions that need to be answered, against a backdrop of the global impact of sepsis. To address this, researchers need to collaborate with colleagues across a diversity of income‐level countries and consider varying research designs.

The importance of addressing post‐sepsis sequelae has been highlighted recently, including targeted therapeutic strategies such as follow‐up clinics where individual impairments in patients could be targeted [3, 14, 15]. Indeed, recent qualitative data from interviews conducted with sepsis survivors on admission to the emergency department and at 3 months following hospital discharge revealed three key themes: new roles in life (where interpersonal relationships were changed as a result of new physical and psychological deficits); cognitive impairment (declining cognitive function, fatigue, lack of executive function); and anxiety (around death, as well as uncertainties and changes to family dynamics) [16]. The authors concluded the importance of tailored post‐hospital discharge management of patients to address this multifactorial decline [16]. Building on this, a recent mixed‐methods study of post‐ICU sepsis survivors and their caregivers identified that nearly three‐quarters of survivors had received rehabilitation, predominantly physiotherapy, with few examples of other interventions for cognitive decline, fatigue or pain [17]. Importantly, participants reported only moderate satisfaction with this therapeutic intervention, highlighting challenges around timeliness, accessibility and specificity as well as improvements needed in structural support frameworks and patient education [17].

These data indicate the perceived gap in service provision to meet these aspects of sepsis recovery and featured within the final top 10 research priorities in our priority setting partnership. However, our findings also reflect how stakeholders rated aspects of the acute stage of patient management to be of crucial importance, including diagnostics, biologics and predictive markers. That the overall top 10 priorities apply across the continuum of patient care underlies the necessity for research that addresses these priorities to be multiprofessional and multidisciplinary in nature to ensure the correct expertise is involved for delivery.

There are multiple strengths to this priority setting partnership. The study benefited from close adherence to JLA methodology, including oversight and chairing by a dedicated JLA advisor as well as comprehensive logistical support in the form of an independent information specialist (KS), dedicated project manager (LB) and governance oversight (CG). Clinical leadership was multiprofessional, reflected by three medical, nursing and physiotherapy experts in critical care; importantly, these leaders remained as non‐participatory observers in the final workshop. Similarly, our steering group brought expertise from relevant and diverse disciplines involved across the continuum of sepsis management and with strong representation from patients and carers. Significant attention was paid to defining the scope of the priority setting partnership from the outset; surveys were then carefully developed, undergoing thorough iteration to ensure clarity of understanding, readability and accuracy to ensure we captured relevant questions to input into the prioritisation process. These surveys were subsequently promoted in a widespread manner via multiple avenues. Finally, in our workshop to produce the top 10 research priorities, we ensured a balance between healthcare professionals and those with lived experience, recognising the equal importance of these stakeholders. Feedback (via empirical comments and evaluation survey) from across all workshop attendees, irrespective of role, was overwhelmingly positive, reflecting the event to be an important and protected space for equal voices and exchange of views.

The UK‐centric focus of this priority setting partnership may be considered a limitation. However, whilst this priority setting partnership will predominantly inform the sepsis research agenda in the context of the UK healthcare jurisdiction, we anticipate our findings may still be applicable to others internationally. We recognise this may be high‐income countries as our process did not account for factors influencing healthcare research delivery in low‐ and/or middle‐income settings. We recognise the potential bias in survey completion, with respondents self‐selecting based on sepsis experience, albeit a key aim of the JLA priority setting partnership process was to engage with those who have lived experience of the topic under investigation as well as those responsible for the delivery of healthcare to these individuals. As the surveys were available publicly, we are unable to determine a denominator (and therefore response rate). However, both our respondent numbers and questions are in keeping with other recent JLA priority setting partnerships [18, 19, 20, 21].

We undertook focused efforts to reach under‐served populations to participate in this priority setting partnership, but we recognise that our recruitment is not fully representative of the UK census. Moreover, we did not collect details of socio‐economic status and cannot confirm whether respondents were representative of the UK population. Further work is needed to ensure the involvement of these populations in the design, delivery and dissemination of future research generated from the findings of this priority setting partnership. Notably, intentional research inclusivity has recently been adopted as a core element of applications to the National Institute for Health and Care Research to address health and care inequalities [22]. Finally, whilst our priority setting partnership scope was inclusive of patients, family members, caregivers and clinicians, we did not include certain populations where the steering group felt dedicated attention was considered more appropriate to meet specific needs, e.g. bereaved family members.

In conclusion, priorities for sepsis research have been established through a rigorous process of consensus involving patients, carers and clinicians. These priorities reflect the continuum of sepsis management, from acute through to survivorship, and will support future delivery of meaningful research to improve outcomes from sepsis. Further work is needed to ensure the involvement of under‐served populations in the design, delivery and dissemination of future research generated from the findings of this priority setting partnership.
